# Assessment of Vedolizumab and UC-MSC interaction and therapeutic potential in acute graft-versus-host-disease

**DOI:** 10.3389/fcell.2026.1836978

**Published:** 2026-05-21

**Authors:** Anna Merlo, Serena Zilio, Martina Bernardi, Daniela Catanzaro, Luisa Galla, Laura Zocca, Olivia Marini, Martina Piccoli, Alberto Tosetto, Weisha Qi, Ilaria Marigo, Francesca Elice, Giuseppe Astori

**Affiliations:** 1 Advanced Cellular Therapy Laboratory, Haematology Unit, Vicenza, Italy; 2 Veneto Institute of Oncology, IOV – IRCCS, Immunology and Molecular Oncology Diagnostics, Padua, Italy; 3 Transplant Program, Haematology Unit, Vicenza, Italy; 4 Department of Surgery, Oncology and Gastroenterology, University of Padua, Padua, Italy

**Keywords:** acute graft-versus-host disease (aGvHD), combination immunotherapy, humanized mouse model, umbilical cord-derived mesenchymal stromal cells (UC-MSCs), Vedolizumab

## Abstract

Acute graft-versus-host disease (aGvHD) is a life-threatening complication of allogeneic hematopoietic stem cell transplantation, characterized by donor T-cell-mediated immune responses against the host. This condition significantly impacts patient quality of life, and it is associated with mortality, particularly when the gastrointestinal tract is affected. This study investigated the potential therapeutic synergy between umbilical cord-derived mesenchymal stromal cells (UC-MSCs), known for their immunomodulatory potential, and Vedolizumab (VDZ), a gut-specific α4β7 integrin antagonist that prevents lymphocyte trafficking to intestinal tissues. Our results demonstrate that UC-MSCs maintain an unaltered immunophenotype and low α4β7 expression in the presence of VDZ, even under inflammatory conditions. Moreover, VDZ exposure did not modify UC-MSC immunomodulatory functions, IDO expression, or susceptibility to peripheral blood mononuclear cell-mediated lysis. In an NSG mouse model of aGvHD, single-agent therapies showed limited effects on survival, whereas combined VDZ and UC-MSCs highlighted the critical importance of optimizing administration windows and dosing schedules. Although no clear synergistic therapeutic benefits were observed in this model, the absence of toxicity or mutual inhibition establishes a favorable safety profile for the combination of VDZ and UC-MSCs as rescue therapy for steroid-refractory aGvHD. These findings also provide a rationale for exploring this combination in a prophylactic setting, preventing gastrointestinal aGvHD by limiting early lymphocyte trafficking while preserving systemic immune regulation.

## 1 Introduction

Acute graft-versus-host disease (aGvHD) is a major complication of allogeneic hematopoietic stem cell transplantation (HSCT), affecting 40%–50% of patients. About 15% develop grades III–IV disease. The response to first-line therapy (high-dose steroids) in severe aGvHD is only 30%–40%. Steroid-refractory lower gastrointestinal (GI) aGvHD has a dismal prognosis, with 40% mortality and a low response rate to the second-line Ruxolitinib therapy (Jamy et al., 2021; Biavasco et al., 2022). aGvHD results from an immune response where donor-derived T-cells recognize and attack recipient tissues, primarily the skin, liver, and GI tract, in response to host antigen presentation, damage-associated inflammation, and chemokine recruitment of effector cells (Zeiser and Blazar, 2017). The multiple pathways involved in this pathogenesis and the unsatisfactory clinical outcome have prompted investigation of drug combinations for the prevention and treatment of aGvHD. However, studies exploring the interactions between these agents are often lacking.

Vedolizumab (VDZ) is a humanized monoclonal antibody that binds exclusively to the α4β7 integrin on pathogenic gut-homing lymphocytes. It selectively inhibits their adhesion to the intestinal mucosal vascular addressin cell adhesion molecule 1 (MAdCAM-1) while sparing vascular cell adhesion molecule 1 (VCAM-1). Unlike VCAM-1, MAdCAM-1 is mainly found in blood vessels within the intestinal mucosa and gut-associated lymphoid tissue. VDZ has no known systemic immunosuppressive effects (Soler et al., 2009). The α4β7 integrin is present on a specific subset of memory T-lymphocytes that tend to migrate into the GI tract and can trigger inflammation associated with ulcerative colitis and Crohn’s disease. By blocking the interaction between α4β7 and MAdCAM-1, VDZ prevents the extravasation of T-lymphocytes from blood vessels into inflamed intestinal tissue (Vedolizumab, 2016). Petrovic and colleagues (Petrovic et al., 2004) demonstrated that α4β7 is a critical homing integrin on alloreactive T-cells in the development of GI aGvHD, and the expression of α4β7 on donor T-cells is essential in developing intestinal GvHD in mouse models (Dutt et al., 2005). Choi and colleagues (Choi et al., 2012) reported that blocking alloreactive donor T-cell trafficking to target organs significantly reduces aGvHD severity in allogeneic HSCT models.

Therefore, inhibiting α4β7-mediated trans-endothelial migration is an appealing strategy for preventing or treating aGvHD. This is further supported by the fact that MAdCAM-1 is constitutively expressed on the endothelial venules of Peyer’s patches, which are vital sites for the development of the anti-host cytotoxic T-cells causing GI aGvHD (Murai et al., 2003).

Accumulating clinical evidence suggests that VDZ is more effective when administered as prophylaxis rather than as treatment for established aGvHD, particularly with GI involvement (Goto et al., 2025). In this context, prophylactic use refers to administration before or during early T-cell activation and gut homing, whereas therapeutic use indicates administration after the establishment of a clinical intestinal aGvHD, when tissue damage and inflammation are already ongoing.

Recently, an international phase III randomized, double-blind trial demonstrated a significant improvement in lower-GI aGvHD–free survival from day +180 when VDZ was added to standard calcineurin inhibitor–based prophylaxis compared with placebo (Chen et al., 2024). In contrast, clinical studies evaluating VDZ as treatment for established, steroid-refractory GI aGvHD have reported heterogeneous and often delayed responses, with reduced efficacy in advanced disease stages. This is likely due to irreversible mucosal injury and the activation of inflammatory pathways independent of lymphocyte trafficking (Floisand et al., 2017; Li et al., 2022; Zeiser et al., 2020). Together, these findings support the concept that VDZ exerts its maximal clinical benefit when used prophylactically, while its therapeutic role may be limited to selected patients with early or less severe GI involvement.

Mesenchymal stromal cells (MSCs) are multipotent progenitor cells capable of self-renewal and differentiation into various mesodermal lineages (Capelli et al., 2011; Erices et al., 2000; Zuk et al., 2002). Umbilical cord-derived MSCs (UC-MSCs) exhibit strong immunomodulatory, anti-apoptotic, angiogenic, and anti-fibrotic properties through paracrine and juxtacrine signaling. Key factors such as interferon-γ (IFN-γ), indoleamine 2,3-dioxygenase (IDO), and nitric oxide synthase (NOS) enable these cells to suppress T- and B-cell proliferation, modulate dendritic cell function, and inhibit NK-cell activation (Krampera, 2011). Furthermore, UC-MSCs are well-suited for combination therapies owing to their safety: they do not raise infection risk, persist long-term, or express HLA class II antigens and co-stimulatory molecules, thus eliminating the risk of exacerbation of aGvHD (Thompson et al., 2020). Their efficacy in treating steroid-resistant aGvHD has been demonstrated in clinical studies [reviewed in (Can et al., 2017)], and interesting findings suggest that MSC-induced immunosuppression may even be mediated by apoptosis following infusion, highlighting a new mechanism behind their therapeutic effects (Galleu et al., 2017).

To lay the foundations for a potential therapeutic protocol combining UC-MSCs and VDZ, we evaluated their potential interaction *in vitro* and *in vivo*. To achieve this, we developed a humanized aGvHD mouse model using human peripheral blood mononuclear cells (PBMCs) to assess the potential therapeutic synergy and safety of this dual-targeting approach.

## 2 Methods

### 2.1 UC-MSC isolation and characterization

After ethical committee approval (Act 111946 dated 5.11.2019, Comitato Etico per le Sperimentazione Cliniche, AULSS8 Berica) and informed consent signature, umbilical cords (UCs) were collected after cesarean section from the Obstetrics of Vicenza and Arzignano General Hospitals. UCs were treated, and cells were isolated and expanded as described in (Astori et al., 2020). After approximately 1 month of culture, the adherent cells were harvested and cryopreserved in liquid nitrogen vapors. UC-MSCs were evaluated for trilineage differentiation into osteogenic, adipogenic, and chondrogenic lineages using specific induction media and standard staining techniques (data not shown).

### 2.2 Immunophenotyping

UC-MSCs were cultured with and without 10 μg/mL VDZ in the culture medium, and characterized according to ISCT criteria (Dominici et al., 2006) through flow cytometry. Briefly, cells were stained with either the MSC Phenotyping Cocktail Kit (REAfinity, Miltenyi Biotec) that includes anti-CD73 FITC, or with anti-CD45 PE-Cy7, anti-CD90 FITC, and anti-CD105 PE antibodies (Beckman Coulter) and isotype-matched controls. 7-AAD Viability Dye (Beckman Coulter) was also added. Staining was carried out according to the manufacturer’s instructions. Samples were then acquired using a BD FACSLyric cytometer, and a minimum of 10,000 events were recorded per sample.

### 2.3 Immunomodulation assay

Both basal and induced UC-MSCs were evaluated for immunomodulatory properties. Inflammatory priming was performed by treating UC-MSCs (INDUCED) with 10 ng/mL rh-IFN-γ-1b (Imukin Clinigen) and 15 ng/mL rh-TNF-α (Peprotech) for 48 h of culture. Peripheral blood mononuclear cells (PBMCs) were isolated from buffy coats of healthy donors. Resting and induced UC-MSCs were seeded in 96-well flat-bottom plates (20 × 10^3^, 10 × 10^3^, and 5 × 10^3^ cells/well) in 100 µL complete medium and were left to adhere to the plastic. To measure proliferation, PBMCs were stained with 5,6-carboxyfluorescein diacetate succinimidyl ester (CFSE; Thermo Fisher Scientific). 10 × 10^4^ CFSE-labeled cells were seeded on the UC-MSC monolayer at different PBMC: MSC ratios, namely, 5:1, 10:1, and 20:1. Cells were stimulated with 0.5 μg/mL of anti-CD3 antibody (Miltenyi Biotec) and 500 UI/mL of recombinant human interleukin-2 (rh-IL-2; Miltenyi Biotec). The experiments were carried out in the presence or absence of VDZ or an irrelevant isotype control (10 μg/mL; Ultra Leaf Purified IgG1 Recombinant Antibody; BioLegend). Cells were co-cultured for 6 days before measuring the corresponding decrease in CFSE fluorescence by flow cytometry. For the latter, PE-Cy7 mAb was used to assess proliferation on gated CD45^+^ cells (Beckman Coulter). At least 50,000 events were acquired on a BD FACS LYRIC cytometer (Becton Dickinson). CFSE analysis was performed by the BD FACSuite software version 1.5, and proliferation was quantified as the percentage of cells undergoing at least 1 cell division.

### 2.4 Ido expression

IDO protein expression was analyzed by Western blot. Briefly, UC-MSCs were seeded at 1,000 cells/cm^2^ on a T75 flask for 7 days; after 5 days, inflammatory cytokines in the presence or absence of VDZ or an irrelevant isotype control were added. Cells were then lysed by adding RIPA Buffer. Anti-IDO mAb (diluted 1:1,000; in TBST–BSA 3%; Abcam) and anti-GAPDH mAb (diluted 1:1,000; in TBST–BSA 5%; Genetex) were used to detect target proteins. Data were analysed with ImageJ software.

### 2.5 Cytotoxicity assay

To perform a cytotoxicity assay, a modified protocol from Chieregato et al. was used (Chieregato et al., 2021). Briefly, about 0.05 × 10^6^ UC-MSCs were co-cultured with 1.5 × 10^6^ or 0.5 × 10^6^ PBMCs to achieve 1:30 and 1:10 MSC: PBMC ratios. Co-culture was performed in 5 mL tubes in a 200 µL final volume of buffer (PBS 5% FBS 5 mM EDTA), in the presence or absence of 10 μg/mL VDZ. As viability control, UC-MSCs and PBMCs were cultured alone in the same conditions. After 4 h of co-incubation at 37 °C, 5% CO_2_, cells were stained with CD45-PE-Cy7, CD105-PE, and 7-AAD. To obtain absolute cell counts, cells were transferred to BD Trucount tubes, and multi-color cell analysis was performed in a BD FACSLyric™ cytometer (Becton Dickinson). Data were analysed by using the BD FACSuite software 1.5. The absolute number of cells was calculated according to the manufacturer’s instructions: absolute cell number = (CD105^+^ CD45^+^ 7AAD^−^/n° beads) x (lot-specific n° beads/volume). Cytotoxic activity was quantified as follows: % vital cells = (n° vital UC-MSCs in co-culture/n° vital UC-MSCs at 4h) x 100.

### 2.6 Mouse strain for aGvHD generation

6–8 weeks old *NOD.Cg-Prkdc^scid^ Il2rg^tm1Wjl/SzJ^* (NSG) mice were purchased from Charles River Italia, housed under specific pathogen-free (SPF) conditions, and provided with autoclaved food and water *ad libitum*. All procedures involving animals were conducted under institutional and national guidelines for the care and use of laboratory animals and authorized by the Italian authority (419/2024-PR).

Clinical aGvHD progression was monitored daily using a multi-parameter scoring system. While weight loss and survival were utilized as the primary objective endpoints for therapeutic evaluation, a composite score encompassing posture, activity, fur texture, and stool consistency was used to define humane endpoints. In this acute model, secondary clinical signs typically manifested rapidly within 24–48 h following the onset of initial weight loss, necessitating humane euthanasia before the development of chronic features such as skin integrity changes.

### 2.7 aGvHD induction in NSG mice and administration of UC-MSCs and Vedolizumab

Before human PBMC transplantation, NSG mice underwent sub-lethal 4 Gy total body irradiation using a cesium-137 irradiator. Six hours post-irradiation, mice received intravenous injections of 10 × 10^6^ PBMCs. For all experiments, PBMCs have been administered via tail vein injection. To assess the therapeutic effects of VDZ, it was administered intraperitoneally at doses of 4 (Palaniyandi et al., 2020) or 0.4 mg/kg, using various schedules: as a single administration on day 8 post-PBMC transfer, and as multiple administrations on days 8, 15, and 22. Additionally, UC-MSCs were administered intravenously at doses of 10^4^, 10^5^, or 10^6^ cells per mouse, following the same scheduling as VDZ. This range was chosen since it covers the doses most frequently reported in GvHD literature (Grégoire et al., 2019; Jang et al., 2014). PBMC engraftment has been evaluated by flow cytometry as the percentage of human CD45^+^ cells found in the blood of the animals at different time points. All antibodies have been purchased at BD Biosciences: PerCP-Cy5.5 anti-human CD45, BV786 anti-mouse CD45, FITC anti-human CD3. Live/Dead fixable stain (ThermoFischer Scientific) was used to exclude dead cells from analysis. The samples were acquired on an LSRFortessa (BD Biosciences) equipped with five lasers and analyzed using FSC Express (*DeNovo* Software).

### 2.8 Statistical analysis

The *in vivo* sample size estimation for the current study was done using a two-tailed T-test for a large effect size (d = 0.95, alpha = 0.05) prior to experimental work. Statistical differences among the groups were assessed using both the Log-rank (Mantel-Cox) test for survival analysis and the ANOVA test, with a Bonferroni correction as a post hoc procedure.

## 3 Results

### 3.1 Evaluation of *in vitro* UC-MSCs and VDZ interaction and impact on UC-MSCs properties

**FIGURE 1:**
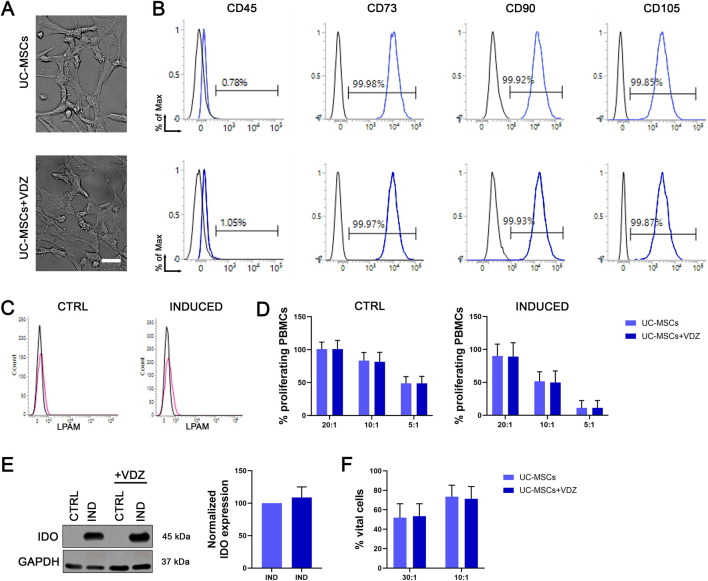
*In vitro* interaction between UC-MSCs and VDZ. **(A)** UC-MSC morphology in standard culture conditions (upper panel) and in the presence of VDZ (lower panel). Scale bar: 20 μm. **(B)** Comparison of the immunophenotype of UC-MSCs cultured under standard conditions and in the presence of VDZ in the culture medium. Isotype controls are shown in black, and the specific antigen signal is colored. **(C)** Expression of α4βt/LPAM on UC-MSCs at basal state (CTRL) or after inflammatory stimulus (INDUCED). Autofluorescence of the cells is shown in black; the specific signal is shown in pink. The figure depicts a representative flow cytometry analysis. We analyzed UC-MSCs from five different donors, at passages three to five in culture; experiments were carried out in triplicate. **(D)** Immunomodulatory activity of UC-MSCs on PBMCs. Percentages of proliferating PBMCs in co-culture with UC-MSCs at different ratios and under various conditions: at baseline (CTRL) and after induction with an inflammatory stimulus (INDUCED), without VDZ (light blue) and with VDZ added (dark blue). No significant differences were found. Experiments involved 5 different batches (UC donors) of UC-MSCs and were carried out in triplicate; the figure shows mean ± SD. **(E)** Expression and quantification of IDO in UC-MSCs under standard (CTRL) and inflamed conditions (IND), in the presence (+VDZ, dark blue) or absence (light blue) of VDZ. No significant differences were found. Experiments involved 5 batches (UC donors) of UC-MSCs and were carried out in duplicate; the figure shows mean ± SD. **(F)** Percentages of viable UC-MSCs remaining after co-culture with PBMCs at two different ratios, in the presence (dark blue) or absence (light blue) of VDZ in the culture medium. No significant differences were found. Experiments involved 5 batches of UC-MSCs and were carried out in duplicate; the figure shows the mean ± SD.

To evaluate the safety and efficacy of a combined treatment between VDZ and UC-MSCs, we verified that there was no direct interaction between the drug and the target cells. First, we performed a standard cell culture, adding the drug to the culture medium of UC-MSCs. The VDZ concentration was chosen to be approximately 20 times higher than that required to saturate VDZ binding sites on whole blood cells (Wyant et al., 2013), enabling the detection of any potential effect of the drug on UC-MSC phenotype and function. Results indicated that the presence of the drug in cultured UC-MSCs did not alter cell morphology, viability, or expression of canonical surface markers, such as CD45, CD73, CD90, and CD105, thus not impacting their immunophenotype (Figures 1A,B; Supplementary Figure S1).

The expression of the VDZ target integrin by UC-MSCs is likely a prerequisite for a direct action of the drug on these cells. To our knowledge, there are no data in the literature on the expression of this molecule on UC-MSCs. In our experiments involving UC-MSCs from five different donors, the integrin α4β7 (LPAM) was found to be expressed at low intensity by a minority of cells in all the batches of tested UC-MSCs. Since the use of VDZ *in vivo* would occur in the context of inflammation, which would exert a certain degree of stimulation on the administered cells, we verified the presence of the drug receptor in UC-MSCs both in basal conditions (CTRL) and after inflammation (INDUCED) and at different passages in culture (from passage three to five) (Figure 1C).

Once the absence of direct interaction was confirmed, we proceeded to verify the maintenance of the standard immunomodulatory and interaction properties of UC-MSCs towards activated PBMCs through co-culture experiments, even in the presence of the drug. The results indicated that VDZ does not affect the immunomodulatory capacity of UC-MSCs since the percentage of proliferating PBMCs remained essentially unchanged compared to the control (UC-MSCs alone) across all experimental conditions using both basal or induced UC-MSCs (Figure 1D).

IDO enzyme is a key molecule for the immunomodulatory activity of MSCs on lymphocytes (Ling et al., 2014). Its expression is induced following an inflammatory stimulus. By Western blot analyses, we quantified IDO enzyme in induced UC-MSCs, confirming that the presence of VDZ does not affect its production or quantity (Figure 1E), supporting the hypothesis that UC-MSCs maintain their standard immunomodulatory mechanisms.

Convincing data (Galleu et al., 2017) support the thesis that UC-MSCs must be lysed by host PBMCs to exert their immunomodulatory activity *in vivo*. We therefore evaluated the susceptibility of UC-MSCs to lysis by PBMCs using a cytotoxicity assay with a cytofluorimetric method established and validated in our laboratory (Chieregato et al., 2021). Again, the presence of the drug did not alter the cytotoxic activity of PBMCs on UC-MSCs, indicating that VDZ does not interfere with *in vitro* properties and activities of the selected cells (Figure 1F).

### 3.2 Therapeutic efficacy of UC-MSCs and VDZ in a GvHD murine model

To investigate the therapeutic potential of UC-MSCs and VDZ in the context of GvHD, survival rates were monitored in irradiated NSG mice following the transfer of human PBMCs.

**FIGURE 2:**
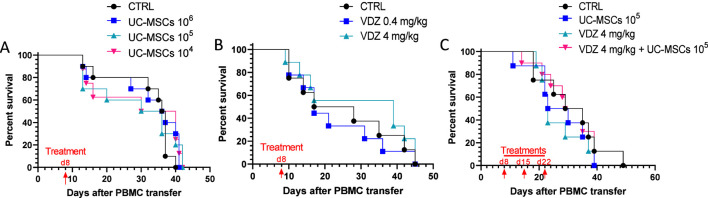
Survival curves of mice with acute graft-versus-host disease under different treatment conditions. After 4 Gy irradiation and transfer of 10 × 10^6^ human PBMCs, NSG mice received different treatments. **(A)** Animals treated with a single administration of the indicated doses of UC-MSCs at day 8 post-PBMC injection (n = 10 per group). **(B)** Animals treated with a single administration of the indicated doses of VDZ at day 8 (n = 8 for CTRL; n = 9 for VDZ 0.4 and 4 mg/kg groups). **(C)** Animals treated with three administrations (at days 8, 15, and 22) of 10^5^ UC-MSCs, 4 mg/kg of VDZ, or a combination of the two (n = 8 for CTRL, MSCs, and VDZ monotherapy groups; n = 10 for the combination group).

Initially, we assessed the impact of a single UC-MSC administration using varying doses (10^6^, 10^5^, or 10^4^ cells) delivered on day 8 post-PBMC injection. Effective humanization and GvHD-associated morbidity (weight loss) were confirmed across all cohorts (Supplementary Figures S2A,B). As shown in Figure 2A, survival rates displayed no significant variation across groups, indicating that a single dose within this range confers negligible therapeutic benefit regarding survival. To mitigate potential donor-specific variability, these data represent pooled results from two independent experiments utilizing distinct human donors.

Similarly, we evaluated the efficacy of VDZ via single intraperitoneal administration of either 0.4 mg/kg or 4 mg/kg on day 8. Within this administration window (day 8 post-PBMC infusion), the use of VDZ should be interpreted as a late prophylactic or early intervention strategy rather than treatment of an established aGvHD as previously reported (Palaniyandi et al., 2020). Therefore, our model does not fully recapitulate a therapeutic setting of an advanced steroid-refractory disease. Consistent with the cell therapy findings, robust engraftment and comparable weight loss were observed (Supplementary Figures S2C,D), and no dose-dependent effects on overall survival were observed. Figure 2B demonstrates that survival outcomes in animals treated with either the high or low VDZ dose were comparable to the control group, suggesting that a single administration does not significantly alter survival trajectories in this setting.

To determine whether a repeated dosing schedule or a combination of the two therapies could elicit a synergistic effect, we implemented a multi-dose protocol. This regimen involved three administrations (on days 8, 15, and 22) of 10^5^ UC-MSCs, 4 mg/kg VDZ, or a combination of both. Despite consistent humanization and typical GvHD manifestation (Supplementary Figures S2E,F), this intensified intervention strategy did not yield statistically significant differences in overall survival among the treatment cohorts or compared to controls (Figure 2C).

Treatment timing also proved critical to overall outcomes. When we evaluated an early-intervention regimen (administrations on days 3, 6, and 9 of 10^6^ MSCs, 4 mg/kg VDZ, or both), we observed decreased survival and accelerated disease progression compared to untreated controls (Supplementary Figure S3). All these data indicate that mono-therapy application or premature immune modulation in this specific model can actually be counterproductive, emphasizing the need to carefully define the optimal therapeutic window.

## 4 Discussion

This work aimed to verify in a preclinical setting whether the combination of VDZ and UC-MSCs, already used individually in the clinic for the prophylaxis and treatment of steroid-refractory aGvHD, could be employed to formulate a new therapeutic protocol.

The use of UC-MSCs in the treatment of aGvHD began several years ago in a pediatric patient (Le Blanc et al., 2004). Over time, results have varied across studies. Recently, UC-MSCs received FDA approval for treating steroid-refractory aGvHD in the pediatric setting, where outcomes were uniformly better and more consistent (Etra et al., 2025; Kurtzberg et al., 2020). In general, UC-MSCs are safe and well-tolerated in adults as well, but several studies and meta-analyses have shown that their efficacy in treating aGvHD is highly variable (Kadri et al., 2023; Kelly and Rasko, 2021). On the other hand, VDZ has been shown to be safe and effective in the prophylaxis of lower-GI aGvHD in adults (Chen et al., 2024; Li et al., 2022) with a well-known mechanism of action involving the interaction with the integrin α4β7 (Luzentales-Simpson et al., 2021; Wyant et al., 2013). Given these promising results, we explored whether combining VDZ and UC-MSCs could enhance aGvHD treatment efficacy or whether UC-MSCs might still be effective as a third-line therapy.

In our *in vitro* experiments, exposure to the drug did not affect UC-MSC identity or stability, as the standard UC-MSC markers remained unchanged. Additionally, integrin α4β7 is expressed only at low levels by a small subset of UC-MSCs. To our knowledge, these findings have not been reported before in the literature and provide new insights into the interaction between VDZ and UC-MSCs, supporting a non-direct mechanism of action of the drug on UC-MSCs. Even under inflammatory stimulation, conditions that better mimic an *in vivo* GvHD microenvironment, integrin expression stayed minimal, further confirming that VDZ does not directly influence UC-MSCs. Moreover, UC-MSCs maintained their immunomodulatory capacity on PBMCs even in the presence of VDZ. The drug did not alter either their immunomodulatory activity or their susceptibility to lysis by PBMCs. This indicates that VDZ can be used in combination with UC-MSCs without compromising their beneficial properties, such as the expression of IDO enzyme or the inflammation-induced apoptosis, mandatory to exploit the full immunomodulatory effect of UC-MSCs (Cheung et al., 2023; Ling et al., 2014).

Humanized mouse models are useful for studying GvHD mechanisms and identifying key molecular targets. However, their translational relevance remains limited, as interspecies differences may affect clinical applicability (Hulsdunker and Zeiser, 2015). Although our *in vitro* results demonstrate that VDZ and UC-MSCs can coexist without direct interference, this did not translate into *in vivo* efficacy. In our humanized NSG-aGvHD model, neither monotherapy nor the combined treatment significantly improved survival.

In this context, our experimental design mainly reflects a prophylactic or early-intervention setting, and this distinction is critical as VDZ is known to be more effective in preventing lymphocyte trafficking than in reversing established tissue damage (Wyant et al., 2013). Therefore, the lack of significant therapeutic benefit observed in our model may be interpreted in light of this biological and clinical context.

Nevertheless, these contrasting outcomes offer a valuable perspective: biological compatibility is a prerequisite for, but not a guarantee of, therapeutic synergy.

Our findings suggest that the therapeutic window for this specific combination is narrower than initially hypothesized. The timing of integrin blockade relative to T-cell expansion likely acts as a decisive variable. While the mechanistic rationale for pairing these agents remains sound, future success will likely require more refined dosing schedules or the integration of standard-of-care prophylaxis (such as calcineurin inhibitors) rather than relying on dual monotherapy. Alternatively, using primed or apoptotic UC-MSCs, as suggested by other groups (Mendt et al., 2021; Amison et al., 2024), may improve the *in vivo* cell function and synergy.

In conclusion, this work defines important boundary conditions for this combination strategy, providing a framework for future translational studies. In particular, they highlight that in combinatorial aGvHD therapy, timing may be as critical as treatment choice. From a clinical point of view, our findings provide several relevant insights. First, the absence of negative interactions between VDZ and UC-MSCs supports the feasibility of this combination strategy. Moreover, our data highlight the importance of treatment timing, suggesting that VDZ may require early administration to achieve optimal efficacy. Finally, although no clear survival benefit was observed in our animal model, the favorable safety profile and biological compatibility of the drug combination support further investigation in clinical settings in earlier stages of the disease.

## Data Availability

The raw data supporting the conclusions of this article will be made available by the authors, without undue reservation.
