# Supplementary material for: Assessment of Vedolizumab and UC-MSC interaction and therapeutic potential in acute graft-versus-host-disease
Source: Front Cell Dev Biol. 2026 May 21;14:1836978. doi: 10.3389/fcell.2026.1836978 (PMC13233507; doi:10.3389/fcell.2026.1836978)
Supplement: Supplementary file 1 [file DataSheet1.docx]

***Supplementary Material***

**Assessment of Vedolizumab and UC-MSC interaction and therapeutic potential in acute graft-versus-host-disease**

**Anna Merlo^1,§^, Serena Zilio^2,§^, Martina Bernardi^1,2^, Daniela Catanzaro^1,2^, Luisa Galla^1^, Laura Zocca^1^, Olivia Marini^1^, Martina Piccoli^1^, Alberto Tosetto^3^, Qi Weisha^2^, Ilaria Marigo^2,4^, Francesca Elice^1,3,*^ and Giuseppe Astori^1^.**

^1^Advanced Cellular Therapy Laboratory, Haematology Unit, AULSS 8 Berica, Vicenza, Italy

^2^Veneto Institute of Oncology, IOV – IRCCS, Immunology and Molecular Oncology Diagnostics, Padua, Italy

^3^Haematology Unit, AULSS 8 Berica, Vicenza, Italy

^4^Department of Surgery, Oncology and Gastroenterology, University of Padua, Padua, Italy

**^§^** These Authors contributed equally to the work

*** Correspondence:**Francesca Elice
francesca.elice@aulss8.veneto.it

# Supplementary Figures


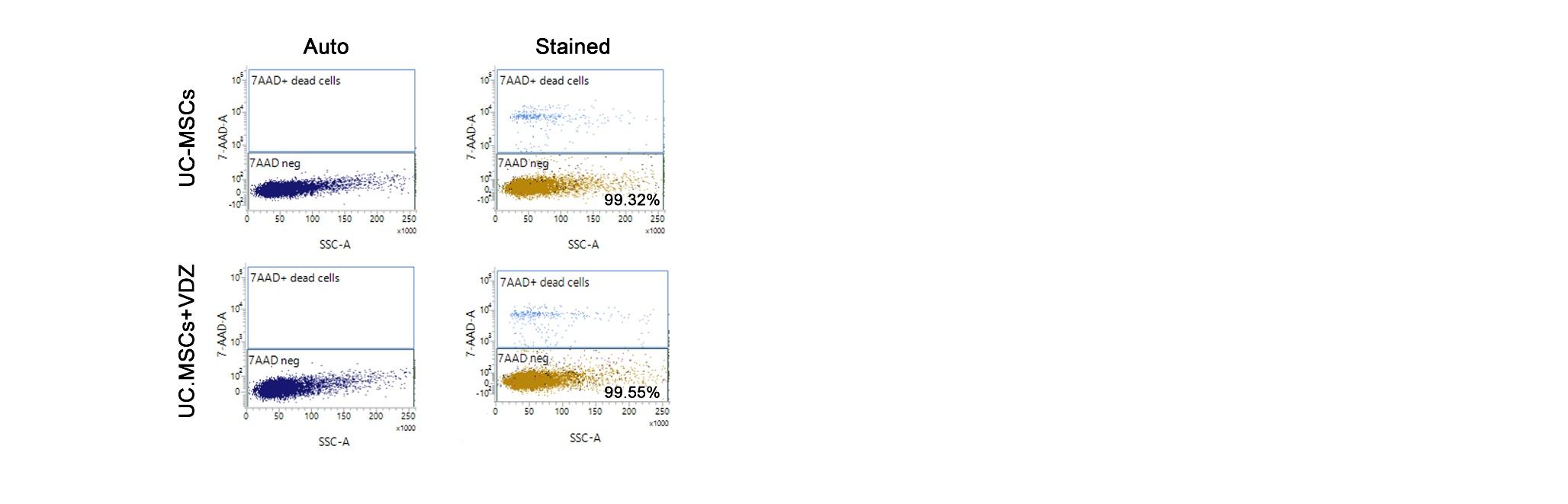


**Figure S1.** ***In vitro* cell viability with VDZ.** Cell viability assessment with and without the addition of VDZ, and the gating strategy for subsequent immunophenotype analyses.


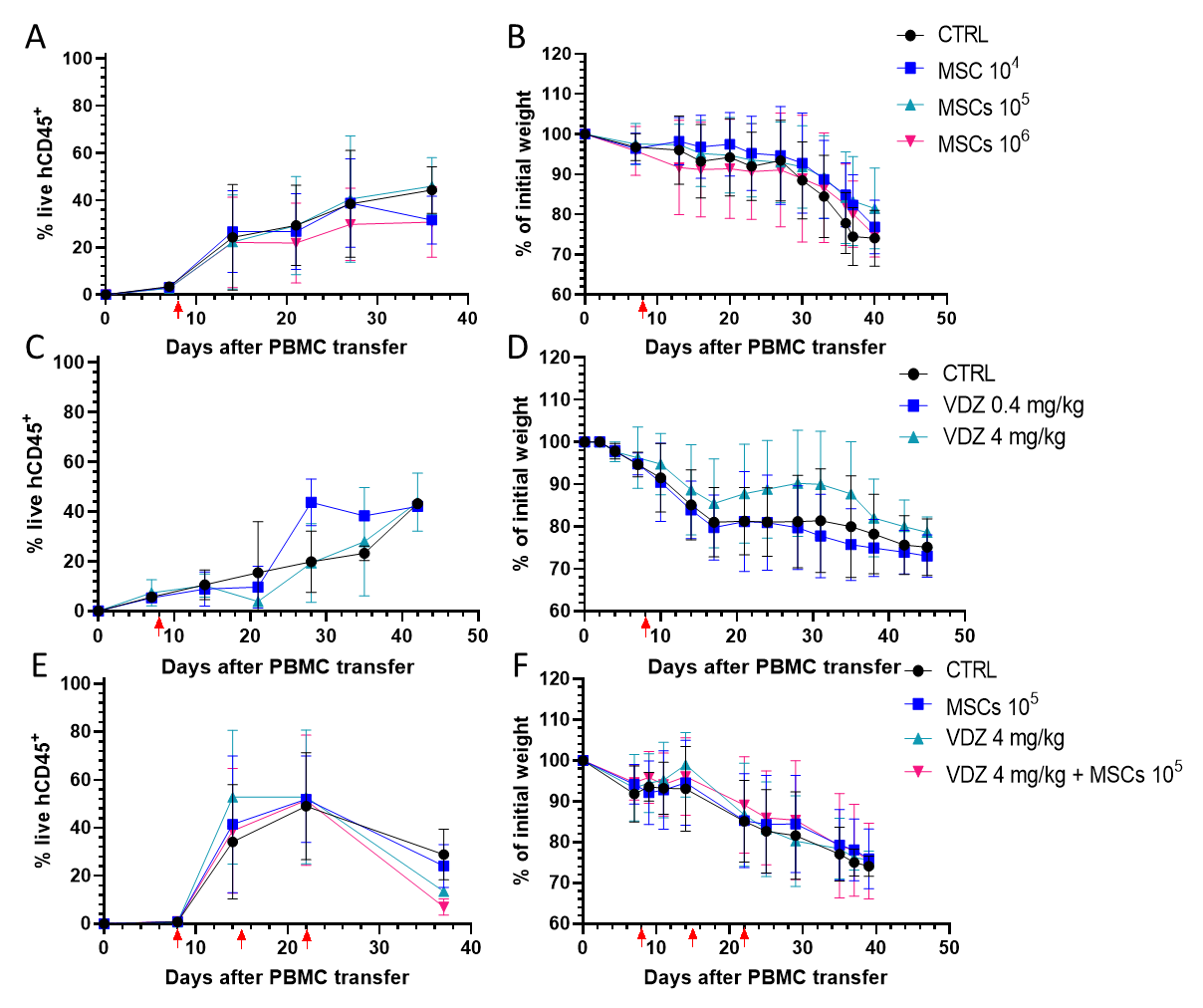


**Figure S2.** **Human immune engraftment and GvHD-associated weight loss kinetics in the NSG model.** The panels depict the percentage of human CD45^+^ cells in the peripheral blood of NSG mice (panels A, C, and E) to confirm effective humanization, alongside weight alteration expressed as a percentage of initial body weight (panels B, D, and F) to monitor GvHD progression. All mice were subjected to 4 Gy irradiation followed by the transfer of 10 x 10^6^ human PBMCs. The data represent three distinct therapeutic evaluations: **A, B.** Animals treated with a single administration of the indicated doses of UC-MSCs on day 8 post-PBMC injection (n=10 per group). **C, D.** Animals treated with a single administration of VDZ at 0.4 mg/kg or 4 mg/kg on day 8 (n=8 for CTRL; n=9 for VDZ-treated groups). **E, F.** Animals received a repeated dosing regimen consisting of three administrations (days 8, 15, and 22) of 10^5^ UC-MSCs, 4 mg/kg VDZ, or a combination of the two (n=8 for CTRL, UC-MSCs, and VDZ monotherapy groups; n=10 for the combination group). All graphs report mean percentages ± SD. Where applicable, red arrows indicate the specific days of treatment administration.


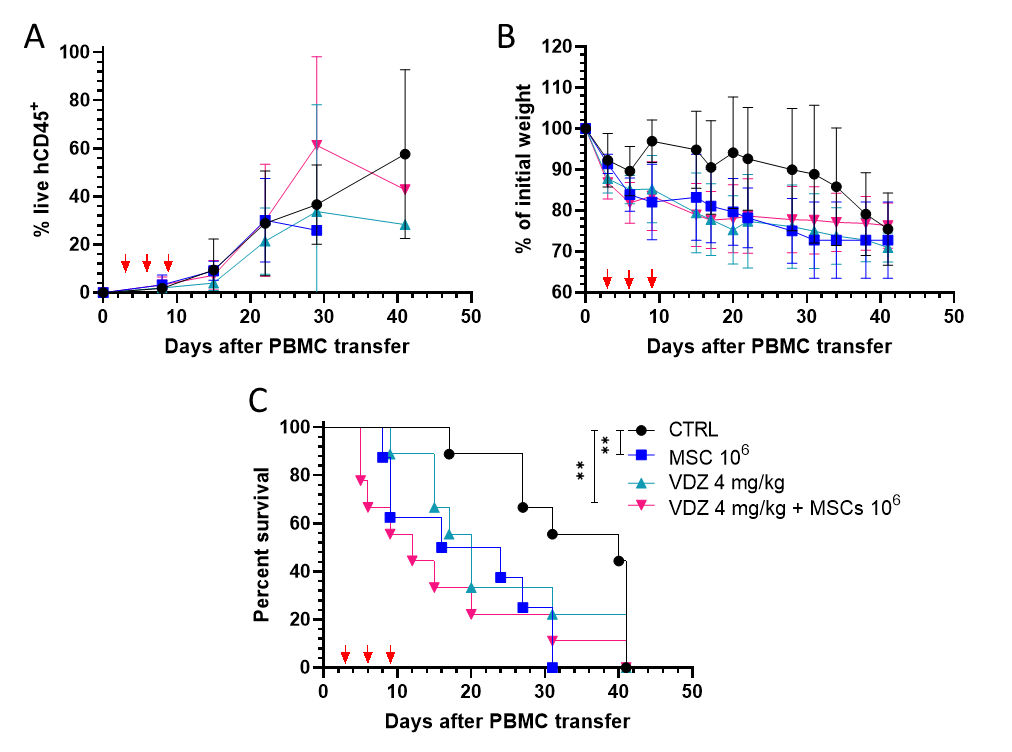


**Figure S3.** **Disease progression and survival in NSG mice following early, repeated administration of MSCs and VDZ.** The panels illustrate the evaluation of GvHD kinetics in animals subjected to 4 Gy irradiation and subsequent transfer of 10 x 10^6^ human PBMCs. Mice received an early multi-dose treatment regimen consisting of three administrations (delivered on days 3, 6, and 9 post-PBMC injection) of 10 x 10^6^ UC-MSCs, 4 mg/kg of VDZ, or a combination of the two. **A.** Percentage of human CD45^+^ cells in the peripheral blood, tracking immune engraftment over time. **B.** Weight alteration, monitored as a percentage of the initial body weight. **C.** Relative survival curves comparing the different therapeutic cohorts and controls. A-B report mean percentages ± SD. For all experimental groups, n=9.
